# The National Awareness and Early Diagnosis Initiative in England: assembling the evidence

**DOI:** 10.1038/sj.bjc.6605382

**Published:** 2009-12-03

**Authors:** M A Richards

**Affiliations:** 1 National Cancer Action Team, St Thomas' Hospital, Westminster Bridge Road, London SE1 7EH, UK

**Keywords:** early diagnosis, cancer awareness, survival, avoidable deaths

## Abstract

A National Awareness and Early Diagnosis Initiative (NAEDI) has been established in England as part of the Government's strategy to improve cancer outcomes. One of the early priorities for this initiative has been to assemble the diverse evidence linking late diagnosis with poor survival and avoidable deaths. This supplement brings together new perspectives on existing research in this area together with findings from recently commissioned research. This paper describes a provisional model, the ‘NAEDI pathway’, for testing hypotheses relating to late diagnosis and its impact. Key findings from other papers in this supplement are also highlighted.

One of the key commitments of the Cancer Reform Strategy (2007) (Department of Health, 2007) in England was to establish a National Awareness and Early Diagnosis Initiative (NAEDI). This initiative was launched in November 2008 and is co-led by the Department of Health and Cancer Research UK, with involvement of a wide range of other stakeholders, including the research community. The Cancer Reform Strategy recognised that excellent progress had been made on early detection of cancer through screening, but more needs to be done to promote early diagnosis in the large majority of patients who present with symptoms.

Members of the NAEDI steering group have identified multiple strands of evidence linking the poor cancer survival rates observed in the United Kingdom in the EUROCARE studies (Sant *et al*, 2003, 2009; Berrino *et al*, 2007) to advanced stage at diagnosis and to delays occurring between the onset of symptoms and the start of treatment. However, the evidence base is complex and is still incomplete.

The aim of this supplement to the *British Journal of Cancer* is to bring together as many of these strands as possible and to present new evidence relevant to NAEDI. It is hoped that this will inform both the future research agenda and the actions of policy makers and commissioners at national and local levels.

## The NAEDI pathway

**Figure 1:**
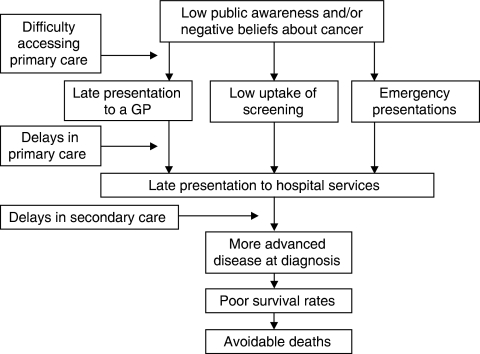
The NAEDI pathway.

To assist thinking about the issues related to late diagnosis of cancer, members of the NAEDI steering group have adopted a provisional ‘NAEDI pathway’ (Figure 1). This should provide a framework for testing various hypotheses regarding late diagnosis and its impact. The first step in the pathway proposes that low awareness of the signs and symptoms of cancer among the public in general, or in specific subgroups, combined with negative beliefs about cancer will lead to late presentation to primary care services and to low uptake of cancer screening services. In addition to this, there may be perceived or actual barriers to accessing primary care services. Ultimately, delayed presentation by patients to primary care services may result in emergency presentations to hospital.

The second step in the pathway involves delays occurring within primary care. These may occur for a variety of reasons, including failure to consider cancer as a possible diagnosis and having inadequate access to diagnostic tests to confirm or exclude cancer as the underlying cause of a patient's symptoms. The difficulties that general practitioners face in this regard should not be underestimated. In England, an average GP will see seven or eight new cases of cancer (excluding non-melanoma skin cancer) each year, but will see hundreds, or possibly thousands, of patients with symptoms that could possibly be due to cancer. When should the GP reassure, observe, request investigations or refer to specialist services?

Delays following referral to specialist services have been well documented in the United Kingdom, with major efforts being made to streamline services to achieve defined waiting time targets. However, relatively little work has been undertaken to measure the relative contributions of patient delay, doctor delay and system delay to overall delay for different cancer sites in this country. Studies of this type have, however, been undertaken in Denmark, another country with survival rates below the European average. A paper in this supplement summarises the findings from Denmark (Olesen *et al*, 2009).

The key hypothesis underpinning NAEDI is that delays lead to patients being diagnosed with more advanced disease and thus experiencing poor 1-year and 5-year survival rates, resulting in deaths that could potentially have been avoided. This could potentially account for at least some of the differences in outcomes observed within the United Kingdom between rich and poor (Coleman *et al*, 2001) and between those from black and minority ethnic populations and Caucasians (Jack *et al*, 2009). It could also account for the differences in survival observed between the United Kingdom and other comparable western European countries.

Although to many people this sequence accords with common sense, with the exception of breast cancer (Richards *et al*, 1999), the linkage between delay and poor survival has been difficult to prove from observational studies. Indeed some studies have reported the apparently paradoxical finding that patients with longer delays may have better survival rates. How might this be explained? In most of the reported studies, the nature of the first symptom has not been reported. It may well be that patients with the most sinister symptoms in terms of prognosis present rapidly to health services, while those with other symptoms may still have early stage disease even after a period of several months. Examples might include abdominal pain and a mass from a right-sided colonic cancer, *vs* rectal bleeding from a more distal cancer – but this still needs to be tested. In patients with breast cancer, it has been clearly shown that long patient delays (and overall delays) are associated with poor survival, while longer doctor delays are associated with better survival (Afzelius *et al*, 1994). The logical explanation for this is that doctors fast track patients with more obvious and advanced breast cancers (Afzelius *et al*, 1994). The paper by Neal (2009) sets out the issues more fully.

## This supplement of the *British Journal of Cancer*

This supplement brings together evidence related to each step on the NAEDI pathway from a diverse range of researchers. These include behavioural scientists, experts in social marketing, public health physicians, primary care academics and epidemiologists.

Stubbings *et al* (2009) describe the development and validation of a new tool to measure public awareness of the signs and symptoms of cancer – the cancer awareness measure (CAM). This tool also includes questions about the barriers to seeking medical help, which may be emotional (e.g. too scared), practical (e.g. too busy) and/or service barriers (e.g. difficult to make an appointment). In the following paper (Robb *et al*, 2009), the first results using the CAM tool are presented. This population-based survey of over 2000 adults showed that awareness of the warning signs of cancer was low when open-ended questions were used. Awareness was lower in those who were male, younger and from low socio-economic status groups or ethnic minorities. In a third paper related to the CAM, the results of using the tool in 1500 men and women from six minority ethnic groups in England are presented (Waller *et al*, 2009). The findings show low awareness across the group as a whole and important differences between ethnic groups, with some striking differences from those shown in the population-based survey using the same tool (Robb *et al*, 2009).

A systematic review of the world literature related to interventions aimed at promoting earlier presentation by cancer patients shows both the paucity of previous research in this field and the lack of evidence-based approaches to promote early presentation for any cancer type (Austoker *et al*, 2009).

In this supplement, two encouraging new approaches to promote earlier presentation are, however, presented. The first describes the development and evaluation of a one-to-one intervention designed to promote early presentation in older women with breast cancer (Linsell *et al*, 2009) – a group where patient delay has been shown to be a particular problem (Ramirez *et al*, 1999). The intervention is being tested among women aged around 70 years who are attending their final routine breast screening appointment. Screening uptake rates in this age group are around 70%, so this approach potentially reaches a large proportion of the older population. The intervention involves a 10-minute interaction with a radiographer. In comparison with women who received usual care or a booklet alone, those who received the interaction and a booklet were more breast aware both at 1 month and 12 months after intervention.

The early findings from a community-based intervention aimed at raising awareness of the signs and symptoms of three common cancers (breast, colorectal and lung) and thus at promoting earlier presentation are presented (Lyon *et al*, 2009). The programme has been tested in subpopulations within Primary Care Trusts with high levels of deprivation. Engagement of local people over the age of 50 years through events in pubs, clubs, mosques, supermarkets and so on is an important component of this programme. Engagement of local GPs is also critical. Preliminary findings are encouraging, especially with respect to the increasing number of cancer cases being referred through the urgent route, though it is too early to be definitive about the impact of the programme (Lyon *et al*, 2009).

The potential for screening to reduce mortality from breast, cervical and bowel cancer has been well established through randomised controlled trials. The effectiveness of a national programme does, however, depend on rates of participation. The factors known to be associated with low participation in screening are reviewed (Weller and Campbell, 2009), along with the evidence of the effectiveness of interventions to promote uptake/coverage. New evidence related to socio-economic inequalities in the uptake of faecal occult blood testing for bowel cancer within London is presented by von Wagner *et al* (2009).

Uptake of national screening programmes for breast and cervical cancer is also known to be low in London. Eilbert *et al* (2009) report on an innovative whole-systems approach to tackle low uptake of breast screening in Tower Hamlets – a deprived area in East London. The approach draws on existing literature about effective interventions to promote breast screening, combined with new analyses to understand the specific problems in Tower Hamlets. A whole-systems approach to improving uptake was then tested. A campaign targeted at Bangladeshi women was undertaken, together with a range of initiatives to promote breast screening through primary care services. The breast screening service itself was also upgraded. Preliminary findings indicate significant improvements both in processes and uptake (which has risen from 44.5% to 58.1%). Further improvements can be anticipated as a result of recently introduced interventions.

The Bangladeshi community in Tower Hamlets has a high rate of tobacco and areca nut usage, leading to an increased risk of oral cancer. Awareness of cancer is also very low in this population. To tackle this, Cancer Research UK has piloted an awareness raising and screening programme using a mobile dental unit (Nunn *et al*, 2009). Over 1300 people attended 1 of 34 screening days (i.e. about 40 people per day), with 74 (5.6%) being referred on to a specialist service. Five patients were diagnosed with dysplasia and a further 28 had potentially malignant disorders (such as leukoplakia). The feasibility of the approach has been demonstrated.

A contrasting approach that also aims to promote awareness and early detection of mouth cancer is reported by Eadie *et al* (2009). This was targeted at people over 40 years from lower socio-economic groups in the West of Scotland. A social marketing approach was adopted, getting people to tell their own stories using mass media. The aim was to increase people's feelings of personal risk, while enhancing feelings of efficacy and control. Assessments conducted after the campaign indicated that awareness of three important symptom features (ulcers, changes that persist and changes to the tongue) was higher in the intervention area than in a control area both at 7 and 12 months.

Research led by primary care physicians into early diagnosis of cancer has grown considerably in strength in recent years. Hamilton (2009) has conducted a series of research studies (the CAPER studies), investigating delays in diagnosis of cancer from a primary care perspective. Importantly, these studies identify the positive predictive value of different symptoms and groups of symptoms. These findings should form the basis for a more rational approach to investigation and referral in the future. The benefits of introducing audits of cancer diagnosis in primary care in Scotland are described by Baughan *et al* (2009). Macleod *et al* (2009) present an overview of what is known about risk factors for patient and GP delay from the world literature.

Two important and complementary analyses based on the EUROCARE-4 data are presented (Møller *et al*, 2009; Thomson and Forman, 2009). These re-emphasise the fact that 1-year survival rates are poor in the United Kingdom across a wide range of cancers. It is widely agreed that, at least for some cancers, poor 1-year survival is a proxy for patients having advanced stage of cancer at diagnosis. Interestingly, among patients who survive for at least 1 year in England, the prospects of surviving to 5 years seem to be closer to the European average. This provides support to the hypothesis that late diagnosis is a significant part of the problem underlying the poor survival rates in this country.

Another important insight into the EUROCARE findings is provided by Abdel-Rahman *et al* (2009), who have estimated how the poor survival rates for individual cancers in the United Kingdom translate into ‘avoidable deaths’. Their analysis indicates that around 11 000 premature deaths per annum might have been avoided if survival rates in England had matched the best in Europe. Against a background of around 125 000–130 000 deaths from cancer each year, this is a very significant figure. Furthermore, around half of these avoidable deaths related to three cancer types: breast, colorectal and lung.

The study by Abdel-Rahman *et al* (2009) does not attempt to quantify what proportion of these avoidable deaths can be attributed to late diagnosis. In the final article in this supplement, this is further explored with particular reference to breast, colorectal and lung cancers (Richards, 2009). We examine the likelihood of differences in screening or treatment (surgery, radiotherapy or chemotherapy) accounting for these avoidable deaths. Although it is impossible to calculate precise figures, we conclude that a large proportion of the avoidable deaths are likely to relate to late diagnosis and to patients therefore not receiving potentially curative treatments. The size of the prize if we can promote awareness and early diagnosis is very substantial.
